# Media Exposure and Post-traumatic Stress Symptoms in the Wake of the November 2015 Paris Terrorist Attacks: A Population-Based Study in France

**DOI:** 10.3389/fpsyt.2021.509457

**Published:** 2021-05-21

**Authors:** Maëlle Robert, Lise Eilin Stene, Dana Rose Garfin, Stéphanie Vandentorren, Yvon Motreff, Enguerrand du Roscoat, Philippe Pirard

**Affiliations:** ^1^Santé publique France, Saint Maurice, France; ^2^Norwegian Centre for Violence and Traumatic Stress Studies, Oslo, Norway; ^3^Sue & Bill Gross School of Nursing, University of California, Irvine, Irvine, CA, United States; ^4^Laboratoire Parisien de Psychologie Sociale, EA 4386, Université Paris Ouest Nanterre-La Défense, Nanterre, France

**Keywords:** terrorism, post-traumatic stress symptoms, mass media, social media, mass casualty incidents, public health, mental health

## Abstract

The intense mass media coverage of the Paris terrorist attacks on November 13, 2015 exposed a majority of the French population to the attacks. Prior research has documented the association between media exposure to terrorism and post-traumatic stress symptoms (PTSS). The present study replicated and extended these findings in a French sample. A population-based sample (*N* = 1,760) was drawn from a national web-enabled panel in June 2016. Hours of attack-related media exposure (i.e., TV-watching, viewing internet images, engaging in social media exchanges) in the 3 days following the attacks were assessed. Multivariate regression models, adjusting for gender, age, direct exposure (i.e., witnessing in person or knowing someone injured or killed), residential area, social support, pre-attack mental health service utilization, and other adverse life events, examined the association between media exposure and PTSS (assessed using the self-report PCL-5). Compared to those reporting less than 2 hours of daily attack-related television exposure, those reporting 2–4 hours (β = 3.1, 95% CI = 0.8–5.3) or >4 hours (β = 4.7, 95% CI = 2.0–7.4) of media exposure reported higher attack-related PTSS. This finding was replicated with social media use: those with moderate (β = 3.2, 95% CI = 0.9–5.5) or high (β = 6.8, 95% CI = 1.9–11.7) use reported higher PTSS than those reporting no use. Subanalyses demonstrated that media exposure and PTSS were not associated in those directly exposed to the attacks. Results highlight the potential public health risk of extensive mass media exposure to traumatic events.

## Introduction

On Friday November 13, 2015, a series of coordinated terrorist attacks occurred in Paris and the city's northern suburb, Saint-Denis. They began with three suicide bombings outside “Stade de France” (the national stadium of France) during an international football match; were followed by several mass shootings in central Paris targeting cafés and restaurants; and ended with a mass shooting at a concert in the “Bataclan” theater. Altogether, 130 persons were killed and more than 400 were physically injured. These attacks had an immediate psychological impact in the general population (1) and occurred 10 months following terrorist attacks targeting the offices of the Charlie Hebdo magazine and a kosher grocery store (2).

The 24-hour media cycle (including television, newspapers, and social media) turns local disasters into national and international tragedies or “collective trauma.” Prior research demonstrates that, following these events, many in the general population are repeatedly exposed to images of brutal violence through widespread media coverage (3). A number of studies following terrorist attacks in the United States demonstrated that this media exposure may have implications for mental health, with post-traumatic stress symptoms (PTSS) as the most commonly assessed outcome (4). PTSS symptoms include the re-experiencing, avoidance, hyperarousal, and cognitive distortions that characterize Post-traumatic Stress Disorder (PTSD), but may be assessed in the absence of Diagnostic and Statistical Manual of Mental Disorders Criterion A (i.e., trauma exposure) for PTSD (5). Positive associations between time spent watching traumatic images on TV or the internet and PTSS have been reported in the general population at 1 month (6), 1 to 2 months (7), 6 months (8) and even years (6, 9) after terrorist attacks. In these studies, associations were observed after statistically controlling for direct exposure; several studies also controlled for pre-attack mental health (6, 10) and individual-level adverse life events (6). After the September 11, 2001 United States terrorist attacks (9/11), specific trauma-related television images (such as watching individuals jump out of the World Trade Center) and PTSS were linked with increased distress one-to-two months following the attacks (3); after the Boston Marathon bombing, exposure to greater graphic images (e.g., blood) was linked with increased distress 6 months post-attacks (8).

In addition to the robust links between media exposure and PTSS, individual-level factors may also help explain variability in outcomes following collective trauma. Demographic indicators that tend to predict increased distress following traumatic events include female gender, low socioeconomic status, and prior mental health diagnoses (11, 12). As such, epidemiological assessments that include information from socio-demographically variable populations are crucial to understanding the link between media exposure to trauma and subsequent psychological distress. Elucidating these vulnerabilities could help identify segments of the populace with risk profiles linked with higher post-event psychological distress.

Individual-level negative life events that occur prior to a collective trauma may also be associated with greater subsequent distress following a future event. Key research on residents of Detroit found that exposure to prior adversity (e.g., violence, injury) was associated with greater likelihood of PTSD following a subsequent individual-level traumatic event (13). After the Boston Marathon bombing, research indicated that prior exposure to collective trauma was associated with increased acute stress in the aftermath of the attacks (14), as was exposure to individual-level adversity that occurred prior to the attacks (15). Similarly, in the aftermath of the 9/11 terrorist attacks, reporting greater number of pre-event life stressors was correlated with increased PTSS and depression (3). A consideration of an individual's adverse experiences prior to a collective trauma may help parse out variability in post-event psychological responses.

Another key factor that may help explain ongoing distress vs. psychological adaption is social support. Social support can help buffer the effects of stressful events and encourage healthy coping (16–18). Prior research on terrorism has demonstrated that those who report lower social support tend to report higher PTSS (19) and PTSD (20). Moreover, in a study of reactions to terrorism in Israel, social support moderated the relationship between exposure and distress; those with higher baseline social support reported less subsequent distress after exposure to rocket fire (21).

A key limitation of prior research on the relationship between media exposure and PTSS is that most studies examining such associations were conducted in the US; more research is needed in other countries to assess the generalizability of inferences. Furthermore, knowledge is needed about the relationships between the use of social media and stress symptoms in the context of terrorist attacks, particularly given the association between specific types of media exposure (e.g., television, online news) and viewing more graphic content (22). Sound stimuli may also differ between traditional media and social media, and influence the likelihood of developing intrusions (23). A review on the topic indicated a need to conduct further studies on media exposure to trauma, with a specific need for studies to be conducted outside of the US and to focus on type of media exposure (e.g., social media such as Facebook and Twitter) (4). While one study was conducted in France following the Paris terrorist attacks (24), that study did not account for pre-attack mental health, prior exposure to trauma, social media exposure (e.g., Facebook, Twitter), or incorporate information on social support.

To address these gaps in the literature, shortly following the 13 November Paris terrorist attacks, the French National Public Health Agency conducted a national study to examine the mental health effects of the attacks. Using those data, the present study sought to examine the relationship between time spent viewing media coverage of the 13 November Paris terrorist attacks on various media sources (television, online news sources, and social media) during the first 3 days after the events and PTSS seven months later.

## Methods

The present study was conducted seven months after the November Paris Attacks (June 2–20, 2016). Overall, 1,000 participants were recruited from all regions of metropolitan France, including 46 from Paris. In order to capture direct exposure to the attacks, an additional 760 participants were recruited among residents of Paris. Hence, the total sample *N* = 1,760) included 806 residents of Paris and 954 residents from the remainder of France. Respondents were recruited to participate in a general health survey that included questions concerning the Paris attacks. The sample was drawn from the national “Brulé, Ville et Associés” (BVA) company web panel, an existing panel of nearly 700,000 persons in France. Appendix 1 presents sociodemographic characteristics of the BVA panel. Initial voluntary recruitment into the BVA panel occurred through multiple sources including banner ads on commercial and non-commercial partner internet websites and e-mails sent to individuals enrolled in marketing-based loyalty programs. To address the potential for selection bias, special effort was made to contact individuals who are often underrepresented in surveys, such as younger individuals and those with lower socioeconomic status. Once enrolled in the panel, panelists participate in approximately one survey every month. By participating in surveys, panelists earn points that can later be transferred into gift certificates.

**Figure 1:**
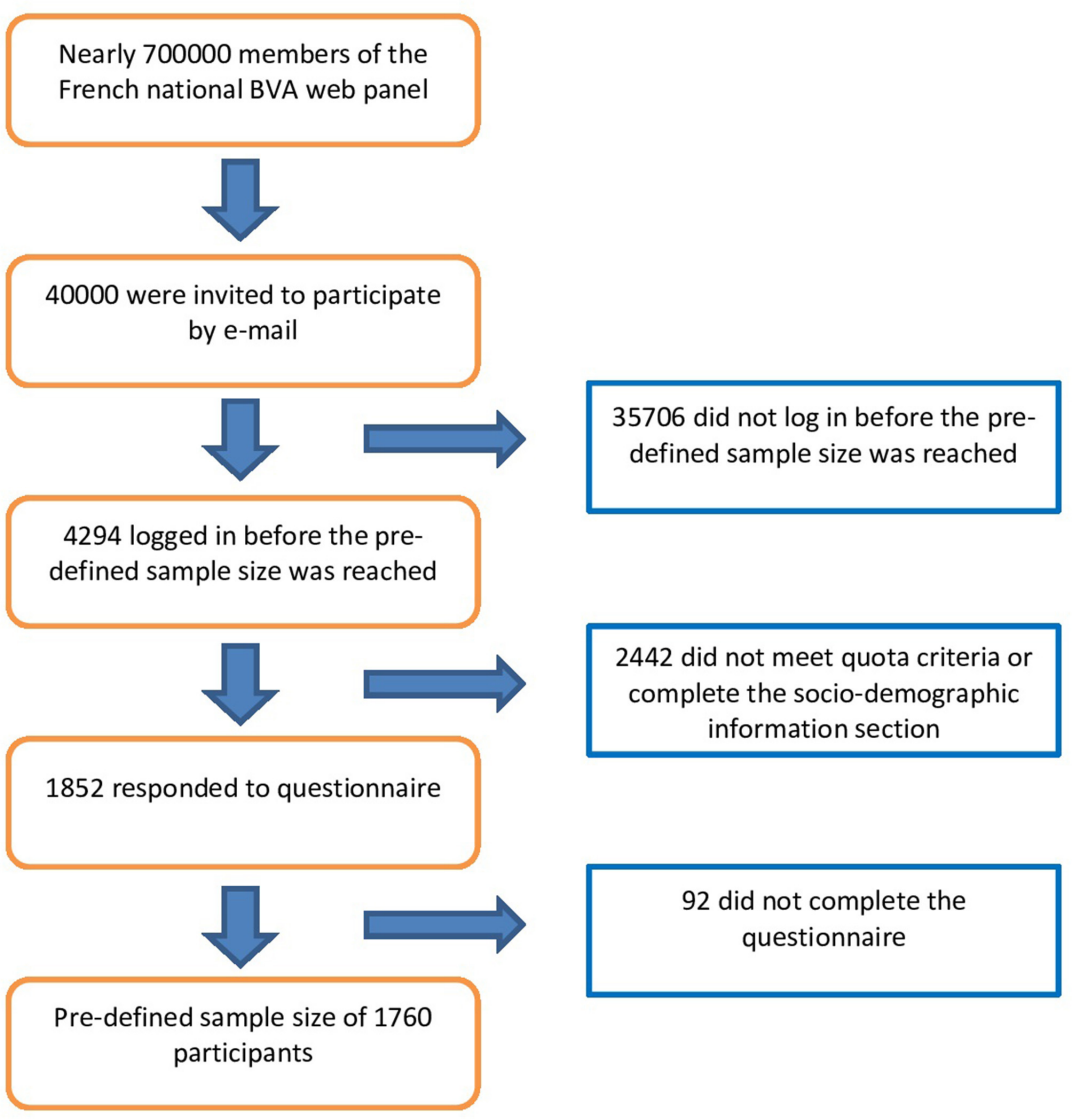
Flow chart of the inclusion of participants in the study.

Figure 1 depicts a flow chart of the inclusion of participants in the study. For the present study, BVA contacted 40,000 panelists *via* email: 25,000 of the invitations were sent specifically to residents of Paris, and the others were sent to residents of all regions of metropolitan France. They were informed the survey would be about health, but not specifically focused on the Paris terrorist attacks. Quota sampling (see Appendix 1 for details of strata per variable) was used to select residents aged 15 years or older according to sex, age (5 strata), socio-professional category (8 strata), administrative region of residence (12 strata) and size of the urban area (5 strata) for residents outside of Paris. Benchmarks were defined according to the 2012 French Census (25). Recruitment stopped when 1,760 people who fulfilled the strata requirements had completed the entire questionnaire. Among the first 4,294 who logged in to answer the questionnaire, 2,442 did not meet quota criteria or did not complete the sociodemographic information section; 92 completed the initial sociodemographic section but not the whole questionnaire. Surveys took ~20 min to complete.

### Measures

#### Event-Related Media Exposure

Respondents were asked to estimate their daily exposure to images of the Paris attacks on TV and the Internet for *each* of the 3 days following the attacks (November 13th, 14th, and 15th): 0 = none at all; 1 = < 1 h; 2 = between 2 and 4 h; 3 = between 4 and 8 h; 4 = 8 h or more. Responses across the 3 days were then averaged and collapsed into: 0 = low media exposure (<2 h per day); 1 = medium media exposure (between 2 and 4 h per day); 2 = high media exposure (more than 4 h per day).

#### Event-Related Social Media Use

Respondents were asked to estimate their use of social media (Facebook, Twitter, etc.) to exchange information about the Paris attacks during the night of the event and over the following weekend with response choices: 0 = none at all (no use); 1 = a little (moderate use); 2 = a lot (high use).

#### Post-traumatic Stress Symptoms

Attack-related PTSS was assessed using the self-report PCL-5 (26). This twenty-item scale assesses the presence of symptoms across the four primary dimensions of DSM-5 PTSD: re-experiencing (Criterion B), avoidance (Criterion C), negative alterations in cognition and mood (Criterion D) and hyperarousal (Criterion E). Symptoms were endorsed on a 5-point Likert-type scale ranging from 0 = not at all to 4 = extremely. Since many participants did not meet DSM-5 Criterion A for direct exposure to trauma they were not assumed to have PTSD. A continuous measure of total number of symptoms was created by summing item scores.

#### Personal Exposure to the Attacks

Participants reported their location the night of the attack. Response options were: (1) Paris, (2) Saint-Denis (a major Paris suburb), (3) other Paris suburb, or (4) elsewhere in France or abroad. Participants who responded that they were in Paris or Saint-Denis answered a follow-up question to determine their precise location: (1) 10th/11th districts or (2) around the football stadium. Individuals who were in a district where attacks occurred were then asked if they had heard gun shots, explosions or people screaming. If they endorsed any of these exposures, they were considered as direct witnesses to the attacks (0 = no direct witness; 1 = direct witness).

Participants also reported whether they knew someone who was killed or injured during the attacks and if so if it was a/an: (1) close friend, (2) close family member, (3) a work colleague, (4) friend of a friend, or (5) an acquaintance.

Based on these responses, a dichotomous measure of direct exposure to the Paris attacks was constructed: 0 = no direct exposure, 1 = self or close friend or close family member directly exposed.

#### Prior Mental Health

Participants were asked the following questions about their pre-attack mental health: (1) *Before November 13th, had you ever taken drugs to solve mental problems or sleeping patterns (tranquilizers, sleeping pill, antidepressants) for a duration of at least 6 months?* (2) *Before November the 13th events had you ever been followed up by a mental health professional for a duration of at least 6 months?* Respondents who answered yes to at least one of these questions were coded as having prior mental health problem; 0 = no prior mental health problem; 1 = at least one prior mental health problem.

#### Adverse Life Events

Adverse life events were assessed by asking participants whether they had ever experienced (1) a trauma before the November 13th attacks (pre-attack trauma yes/no); or (2) a non-traumatic difficulty after the attacks (recent adversity yes/no). Trauma was defined as “*an event which made you feel threatened or on alert for your life: wound, serious illness, physical or sexual violence, rape, natural disaster, war context, etc.”* A non-traumatic event was defined as “*a difficult situation in your personal or professional life: illness, loss of a close relation, divorce, break up, layoff*, *etc*.”

#### Social Support

Social support was assessed by asking about the number of people the participants could count on in case of serious personal matters: (0) none, (1) 1 or 2, (2) 3 to 5, and (3) more than 5. Responses were dichotomized into 0 = none; 1 = could count on at least one person.

#### Demographics

Upon enrollment in the study, gender, age, region of residence, education level, occupational status and household composition were recorded.

### Ethics

Ethical review and approval was not required for this study on human participants nor was written informed consent from the participants' legal guardian/next of kin, in accordance with the local legislation and institutional requirements. This study is based on data collected directly from the participant and automatically anonymized by a polling and survey institute (BVA). According to the French data protection law, it did not need an authorization from the French data protection Authority (Commission nationale informatique et libertés, CNIL). In 2016, when this study was launched, according to French law, only biomedical research needed the approval of an ethics committee. This study did not comply with the criteria required to be qualified as a biomedical research. Therefore, it was not compelled to be reviewed and approved from an ethics committee which would have refused to review the protocol. This has been confirmed by the French ethics committee “Comité de protection des personnes Sud-Est 1” (CPP Sud Est 1). Santé publique France (the French National Public Health Agency) is responsible for the ethical conduct of the study.

### Statistical Analysis

Post-stratification weights were calculated using 2012 French Census benchmarks for age, sex and employment status; region of location and size of the urban area were also included in the weights for the national sample. Initially two sets of weights (one for Paris and one for the remainder of the country) were constructed to account for the oversampling in Paris. The final study weights used information from the calculation of weights from both Paris and the national sample so that analyses could include the entire sample. Unless noted all analyses were conducted using post-stratification weights.

Descriptive statistics were calculated for the entire sample for demographic characteristics, PTSS, media exposure, social media use, prior mental health problems, adverse life events and social support. *T*-tests and *X*^2^ tests were used to compare the individuals' characteristics according to the time spent looking at images *via* media and social media uses. Associations between PTSS and media exposure variables (images broadcasted on TV/Internet and social media use) were examined using a series of Ordinary Least Squares (OLS) regressions. First, bivariate regressions were conducted between PTSS and each independent variable. Next, multivariate models were constructed using a hierarchical variable entry strategy. Variables were entered in the following theoretically meaningful blocks: (1) event exposures (media images on TV/Internet, social media use, direct exposures), (2) individual-level stressors (prior mental health problems, adverse life events), and (3) individuals characteristics (sociodemographic, living location, social support). Lastly, a Wald-test was used to examine potential interactions between (1) media exposure and direct exposure; (2) media exposure and pre-event trauma exposure; (3) social media use and direct exposure and (4) social media use and pre-event trauma exposure. Finally, we did *post-hoc* analyses to examine the bivariate relationships between PTSS and media stratified by direct exposure and pre-event trauma exposure.

## Results

### Event-Related-Media-Exposure and Social-Media Use

Over the 3 days following the attacks, half of the respondents reported watching images on TV or the Internet <2 h per day, 27% reported 2 to 4 h per day, and 22% reported more than 4 h per day. Over the 3 days following the attacks, 66% of respondents reported no event-related social media use, 27% reported low use, and 7% reported high use.

### Sample Description and Bivariate Relationships

Table 1 presents characteristics of the total sample and the respondents' characteristics by event-related media exposure and social media use. Living in Paris or Saint-Denis and experiencing at least one pre-event trauma was associated with moderate or high media exposure (all *p*s < 0.05). Younger participants, women, those living in Paris or Saint-Denis, those with direct exposure to the event, those with pre-event mental health problems, and those with pre-attack traumatic events reported more event-related social media use (all *p*s < 0.05).

**Table 1:** Sociodemographic, exposure and mental health characteristics in the total sample, and by levels of event-related media exposure and social media use.

	**Total sample (*N* = 1,760)**	**Daily event-related media exposure^a^**				**Daily event-related social media use^a^**			
		**Low exposure: <2 hours per day (*n* = 816)**	**Medium exposure: 2-4 h per day (*n* = 518)**	**High exposure: >4 h per day (*n* = 426)**		**No use (*n* = 1,088)**	**Moderate use (*n* = 516)**	**High use (*n* = 156)**	
	**% or mean ± SD**	**% or mean ± SD**	**% or mean ± SD**	**% or mean ± SD**	***P***	**% or mean ± SD**	**% or mean ± SD**	**% or mean ± SD**	***P***
**Sex**					0.99				0.04
Male	47.7	47.7	47.7	47.8		50.7	41.8	43.0	
Female	52.3	52.3	52.3	52.2		49.3	58.2	57.0	
**Age**	46.8 ± 17.3	47.3 ± 16.6	46.0 ± 18.0	46.7 ± 17.6	0.62	50.1 ± 16.3	41.0 ± 17.2	38.9 ± 17.3	<0.0001
**Education level**					0.30				0.30
< High school diploma	28.9	31.3	27.4	25.5		30.3	27.5	21.4	
High-school diploma	21.6	20.1	20.8	26.0		20.0	25.1	23.1	
> High school diploma	49.5	48.7	51.7	48.5		49.7	47.4	55.5	
**Living in Paris or Saint-Denis**					0.02				<0.001
No	93.8	95.3	91.4	93.5		95.4	90.4	92.6	
Yes	6.2	4.8	8.6	6.5		4.7	9.6	7.5	
**Direct exposure**					0.11				
No	94.2	95.5	93.9	91.6		97.0	88.4	90.2	<0.0001
Yes	5.8	4.5	6.1	8.4		3.0	11.6	9.8	
**Prior mental health problems^b^**					0.27				0.03
No	82.4	84.0	82.0	79.0		84.7	77.2	79.8	
Yes	17.7	16.0	18.0	21.0		15.3	22.8	20.2	
**Pre-attack trauma**					0.01				0.01
No	67.5	72.1	62.8	62.9		70.0	65.2	53.7	
Yes	32.5	27.9	37.2	37.1		30.0	64.8	46.3	
**Lack of social support**					0.82				0.09
No	93.7	93.5	93.5	94.6		94.3	94.0	87.5	
Yes	6.3	6.5	6.5	5.4		5.7	6.0	12.5	

*The percentages and means are based on estimates that have been weighted according to the composition of the population of France in 2012*.

*SD = Standard deviation. T-tests were used for continuous variables; chi-squared tests were used for categorical variables. Numbers may not always sum up to 100% due to rounding*.

a *Estimated over a three day period (from Friday evening the 13th to the end of the weekend)*.

b *Prior mental health problems: Having taken drugs or having been followed up for at least six months due to mental health problems*.

### Media Exposure to the Paris Attacks and PTSS

Mean score for the PCL was 17.8 (SD = 16.9). Bivariate relationships between PTSS and media exposure variables are presented in Table 2. Greater exposure to images via TV/Internet and event-related social media use were both positively associated with PTSS. Multivariate analysis indicated that event-related media exposure and social media use were positively associated with PTSS. As presented in Table 3, results remained robust after adjusting for direct exposure (see Model 1), other stressors (i.e., prior mental health problems, adverse experiences; see Model 2), and individual characteristics (i.e., demographics, social support, geographic location; see Model 3).

**Table 2:** Bivariate relationships between post-traumatic stress symptoms (PTSS), media exposure, and social media use (*N* = 1,760).

	**PTSS estimated means**	**β (95% CI)**
**Media exposure^a^**		
Low exposure (<2 hours per day)	15.1	-
Medium exposure (2–4 hours per day)	19.1	4.0 (1.5, 6.5) ^**^
High exposure (>4 hours per day)	22.3	7.2 (4.4, 10.0) ^***^
**Social media use^a^**		
No use	15.2	
Moderate use	21.8	6.5 (4.1, 9.0) ^***^
High use	26.7	11.5 (6.4, 16.6) ^***^

*CI = confidence interval*.

*Estimated means are those calculated from predictions of each bivariate ordinary least squares regression model constructed*.

*β correspond to coefficients estimated by each model*.

a *Estimated over a three day period (from Friday evening the 13th to the end of the weekend)*.

** *p < 0.01*

*** *p < 0.001*.

**Table 3:** Multivariate relationships between post-traumatic stress symptoms (PTSS), media exposure, and social media use (*N* = 1,760).

	**Model 1**	**Model 2**	**Model 3**
	β (95% CI)	β (95% CI)	β (95% CI)
**Event exposure**			
**Media exposure** (mean hours per day)^a^			
Low exposure (reference group)		-	-
Medium exposure (2–4 hours)	3.4 (1.0, 5.7)^**^	3.1 (0.8, 5.4)^**^	3.1 (0.8, 5.3)^**^
High exposure (>4 hours)	5.0 (2.2, 7.9)^***^	4.5 (1.8, 7.2)^***^	4.7 (2.0, 7.4)^***^
**Social media use^a^**			
No use (reference group)	-	-	-
Moderate use	4.9 (2.5, 7.3)^***^	3.8 (1.5, 6.2)^***^	3.2 (0.9, 5.5)^**^
High use	8.7 (3.4, 13.9)^***^	7.9 (3.1, 12.6)^***^	6.8 (1.9, 11.7)^**^
**Direct exposure**			
No (reference group)	-	-	-
Yes	13.7 (8.9, 18.5)^***^	12.2 (8.0, 16.5)^***^	11.4 (7.2, 15.6)^***^
**Other stressors**			
**Prior mental health problems**			
No (reference group)		-	-
Yes		8.6 (5.8, 11.3)^***^	8.6 (5.9, 11.4)^***^
**Pre-attack trauma**			
No (reference group)		-	-
Yes		1.1 (−0.9, 3.1)	1.8 (−0.2, 3.8)
**Recent adversity**			
No (reference group)		-	-
Yes		5.1 (2.7, 7.4)^***^	5.1 (2.8, 7.5)^***^
**Individual characteristics**			
**Sex**			
Male (reference group)			-
Female			1.5 (−0.4, 3.5)
**Age**			−0.04 (−0.09, 0.02)
**Education level**			
< High school diploma			-
High school diploma			−1.1 (−4.0, 1.9)
> High school diploma			−3.6 (−5.9, −1.2)^**^
**Living in Paris or Saint-Denis**			
No (reference group)			-
Yes			2.0 (−1.0, 4.9)
**Lack of social support**			
No (reference group)			-
Yes			8.1 (3.9, 12.4)^***^

*CI = confidence interval*.

a *Event-related media exposure and social media use were estimated over a three-day period (from Friday evening the 13th to the end of the weekend)*.

** *p < 0.01*

*** *p < 0.001*.

*^β^Correspond to coefficients estimated by each model*.

In *post-hoc* analyses of the bivariate relationships between PTSS and media, stratified by direct exposure and pre-event trauma exposure, the positive relationship between PTSS and media exposure remained statistically significant for individuals not directly exposed to the event (see Table 4A). After stratification by pre-attack trauma exposure, the positive relationship between media exposure and PTSS remained robust for both those reporting a prior life trauma and for those not reporting one (Table 4B). However, for individuals not reporting a prior life trauma, only the highest levels of media exposure were associated with higher PTSS. In contrast, for individuals reporting a prior life trauma, both medium and high media exposure were associated with increased distress.

**Table 4A:** Bivariate relationships between post-traumatic stress symptoms (PTSS) (PCL5 score) and media exposure, stratified by direct exposure to the Paris attacks.

	**Not directly exposed to the event (*****n*** **=** **1,624)**		**Directly exposed to the event (*****n*** **=** **136)**	
	**PTSS estimated means**	**β (95 % CI)**	**PTSS estimated means**	**β (95 % CI)**
**Media exposure**				
Low exposure (Reference group) <2 daily hours	14.3	-	28.7	-
Medium exposure2–4 daily hours	17.1	2.8 (1.0,4.6)^**^	27.3	−1.4 (−9.2,6.3)
High exposure> 4 daily hours	21.4	7.1 (5.2,9.0)^***^	28.6	−0.2 (−8.0,7.6)

**Table 4B:** Bivariate relationships between PTSS (PCL5 score) and media exposure, stratified by lifetime traumatic event.

	**No reported lifetime trauma (*****n*** **=** **1,189)**		**Reported lifetime trauma (*****n*** **=** **571)**	
	**PTSS estimated means**	**β (95% CI)**	**PTSS estimated means**	**β (95% CI)**
**Media exposure**				
Low exposure (Reference group) <2 daily hours	14.3	-	17.7	-
Medium exposure2–4 daily hours	15.5	1.3 (−0.8,3.4)	22.4	4.7 (1.4,8.0)^**^
High exposure> 4 daily hours	21.3	7.0 (4.8,9.3)^***^	23.3	5.6 (2.2,9.1)^***^

*CI = confidence interval*.

*Estimated means are those calculated from predictions of each bivariate ordinary least squares regression model constructed*.

*β correspond to coefficients estimated by each model*.

*Event-related media exposure was estimated over a three day period (from Friday evening the 13th to the end of the following weekend)*.

** *p < 0.01*,

*** *p < 0.001*.

## Discussion

Our study presents new data regarding the relationships between media exposure, social media use, and PTSS across France following the Paris terrorist attacks of November 2015. Seven months after the event, we found positive associations between PTSS and both event-related media exposure (number of hours per day watching TV and Internet) and social media use. Furthermore, results indicated that the association between time spent engaging with event-related images and PTSS was primarily relevant for individuals who had not been directly exposed to the terrorist attacks. Results were robust for those who reported prior trauma and for those who did not, although experiencing a prior life trauma appeared to amplify the association between event-related media exposure and PTSS.

Our findings both align with and extend results from prior population-based studies of terrorist attacks. Following the Boston Marathon bombing, a positive association was found between time spent engaging with media content (including television, radio, print, online, and social media) and acute stress scores in covariate-adjusted models accounting for prior mental health, demographics, and direct exposure to the event (10). However, the present study extends those findings by differentiating by type of media and specifically examining event-related social media use. We replicate findings from a study of New York City residents 2 months after 9/11, where relationships were reported between the amount of time spent viewing event-related images and PTSS after adjustment on age, sex and direct exposure (7). Our findings also echo longitudinal work conducted in the aftermath of the Boston Marathon bombings, which found that individual-level stressors and trauma as well as prior mental health problems were associated with PTSS 6 months post attacks (15). We extend those findings by similarly accounting for prior individual-level adversity and by differentiating types of media-based exposure. Finally, our work aligns and extends that conducted in the aftermath of Superstorm Sandy, where a positive association was reported between the use of social media networks and PTSS (27). The present analyses bolster that finding, as we found a similar effect even after including a larger range of potential explanatory variables (e.g., social support, individual-level trauma exposure).

Recent research has examined the impact of stress related to the November Paris attacks in the general French population (24, 28). Positive relationships were observed between PTSS and using traditional media or social media as the major source of information, after accounting for sex, age, geographic proximity and religiosity. Positive relationships were also found between amount of time spent using traditional or social media after the attacks and insomnia symptoms, after adjusting for demographics, location and PTSS. Our study corroborates those findings and also accounts for a larger number of potentially important covariates (e.g., adverse life events, social support, prior mental health problems).

There may be several plausible explanations for our findings. First, general exposure to collective events on traditional media and social media networks may constitute a type of “trauma” that leads to the development of PTSS over the lifespan. Indeed, prior research has shown that exposure to many traumatic events *via* the media may portend more distress over time (14). Many television programs use a dramatic tone to broadcast shocking images in a continuous loop. This is commonly referred to as the “amplification phenomenon,” and can create a climate of stress and fear (29, 30). During and after such exposures, individuals become passively engaged with images then keep watching them repeatedly. Indeed, French television channels covered the events surrounding the Paris terrorist attacks extensively. Yet a distinction can be made between the traditional French TV channels and newer TV channels that focus exclusively on news 24/7. From November 13 to November 20, traditional TV channels devoted, on average, 14% of their programming to covering the terrorist attacks (31). Yet BFMTV, a 24/7 news channel, devoted all its continuous new programing to the events on November 14. On November 13, BFMTV had an audience share of 14%, further highlighting the substantial media-based exposure many in France had to the attacks and corroborating self-report metrics.

Importantly, prior research has indicated that media exposure to collective trauma and psychological distress may interact in a bidirectional, reciprocal relationship (32). Individuals who may be more distressed by images may subsequently and concurrently be more inclined to seek out media exposure (32). It has been hypothesized that individuals who experience distress are more likely to consume media to access information in an attempt to mitigate the feelings of uncertainty associated with collective trauma (33). More longitudinal research is necessary to fully parse out this relationship.

Social media networks operate in a more interactive fashion than traditional mediums. Individuals may exchange freely on a topic, question a situation, and share both diverse emotions and potentially contradictory messages. In this manner, social media may create a type of “contagion phenomenon” in which feelings are spread through social media networks (34). Following a collective trauma, anxiety and stress-eliciting messages are expressed and potentially increase such feelings in online communities. Yet it is plausible that watching event-related images or exchanging on social networks may be a way to cope with stress and feel connected to others in the community during times of stress (35, 36). Future research should further explore this relationship.

Importantly, our study took a fine-grained approach to analyzing the interaction effects between individual-level characteristics, media exposure and PTSS. For those reporting a pre-event trauma, a significant association between time spent viewing attack-related media and PTSS was observed at lower levels of media exposure. This is in alignment with the stress sensitization effect (37), whereby previous stressors portend more intense responses following a subsequent adverse event. Indeed, prior work suggested that a decreased capacity to deal with fear, evident at the neurobiological level, may be a component of PTSD development (38, 39).

In our study, the positive relationship between “traditional media” exposure and PTSS was only observed for individuals indirectly exposed *via* the media: this relationship was not evident for those with direct, Criterion A trauma exposure to the attacks. Our work contrasts with a study of a sample of New York residents after 9/11, which found that frequently viewing TV images was associated with PTSD among those who were directly impacted by the attacks, but not among those not directly impacted by the attacks (3). However, that study was conducted 2 months after the 9/11 events and among a sample of Manhattan residents.

Our study found no interaction effect between event-related social media networks use and direct exposure with regard to PTSS symptoms, suggesting that social media use and television watching may function differently among those directly exposed to a trauma. In the immediate aftermath of a collective trauma, social media use also may be a way to connect and assure the safety of friends and loved ones. In fact, prior research suggested the availability of social support was associated with lower PTSS after trauma exposure (40). Our study indicated that younger people and women had a higher event-related use of social media than older individuals and men. A study conducted in France the same year as the terrorist attacks also documented that younger participants generally used social media more often than older participants (41, 42). The same study observed differences in the way women and men used social media: women were more likely than men to use social media to share feelings and to keep or develop social contacts. The need for sharing feelings and seeking connectedness might be particularly high in the wake of a collective trauma. This may explain why women were more inclined to engage on social media after the terrorist attacks (43).

It is important to note that the level of PTSS scores was two times higher for individuals directly exposed compared to those not directly exposed. Our results suggest that direct exposure overshadowed the effect of media exposure 7 months following exposure. Of note, media exposure to the attacks does not satisfy the trauma criterion A of a PTSD diagnosis according to the DSM 5 (5). Moreover, the “directly exposed” category in our study includes different types of direct exposure (e.g., personally in attacks, hearing about the attacks, having relatives in the attacks) that may have qualitative differences. Given the small sample size of those directly exposed, teasing apart these potential differences was beyond the scope of the present analyses. Moreover, exposure was collected retrospectively 7 months after the attacks and thus may be subject to recall bias, whereby only the most impactful exposures may have been reported. Lastly, individuals with the most severe exposure may have been unable to watch images on TV or exchange information on social networks as they were in the throes of the crises. Indeed, prior research on school shooting suggests that the type of information being shared on social media during a collective trauma has implications for subsequent traumatic stress responses (44). Future research should explore this relationship further in the context of a terrorist attack.

Our study has several limitations. First, we selected our sample from a pre-existing web panel using quota-based methods. It is possible that individuals who decided to take the survey differed from those who did not, causing selection bias. Second, our study was cross-sectional and thus precludes causal inferences. Our reports of media exposure in the days after the Paris attack were collected retrospectively and thus may be subject to recall or misreporting bias. Both under- and overreporting may occur in self-reported media use (45). While we assessed exposure to media coverage of the attacks on TV and online sources, we did not ask about listening to the radio or reading newspapers. We did not have information about the amount of time the respondents used media in general, outside the context of the terrorist attacks. Therefore, it is unknown whether the factors associated with media use in our study were specific to the context of the terrorist attacks, or whether they represented variability in media use more generally. This information should be collected in future research. While we sought to include a wide range of potential confounding factors, we did not control for all possible confounds (e.g., pre-event TV habits). Moreover, our life trauma and pre-mental health measurements were proxy measures that may have lacked the precision of a clinical assessment. Similarly, since our panel was web-based, it is possible that those who took the survey used social media networks after the event more than those who were not on the panel. In order to address the limitations of the current study, future studies should include a sample randomly selected from the general population rather than from a pre-selected opt-in web-panel. Furthermore, longitudinal assessments should be made, and include pre-event measurements. New technologies (e.g. smart phones) may provide more objective and detailed measures of media utilization. More in-depth information about the participants' media exposure and associated psychological distress should also be obtained through interview-based studies and qualitative analyses.

Despite our study's limitations, the results highlight the need to address the potential deleterious impact of media exposure on psychological distress in the general population. Special attention should also be provided for children, as it was not done in this study and children are also likely vulnerable to such exposure (46–48). More generally, there is a lack of knowledge on measures to efficiently prevent and mitigate PTSS symptoms after media exposure to terrorist attacks and similar mass casualty incidents. Recent research indicates that there may be a “cycle of distress” where people with higher PTSS before an attack are more likely to experience an increase in PTSS when they are exposed to media coverage of the attacks (32). It may therefore be beneficial to inform the general public about the possible stressful effects of repeated or prolonged media exposure to the attacks, particularly if one experiences psychological distress or has prior psychological disorders. With respect to traditional media, a recommendation for public health officials and media reporters and executives would be to engage in in-depth reflection on the media coverage of such events (49–51). One main question would then be how to relay essential information in the context of a terrorist attack without eliciting undue additional distress. Prior work suggests that some images are more likely than others to elicit PTSD (3); thus limiting certain images in media reports could be one way to reduce the potential for amplifying post-event distress in the populace. Key questions to consider include: which images should be disseminated? Should we recommend warning messages like banner ads to alert on the fact that some images are shocking, or that watching them in a continuous loop during a long time might produce adverse psychological effects? This type of work has begun in France with journalism students, by encouraging students to consider the best way to relay information about suicide in the media (52). With respect to social media networks, the solution may be more complex because regulation is less standardized. However, similar reflection across the companies managing these platforms may be useful as well.

Our study demonstrated, using a population based sample in France, that media exposure in the immediate aftermath of the 2015 Paris terrorist attacks left an indelible mark on the general populace. Seven months after the event, many across France reported high levels of event-related PTSS, which was linked with high levels of media exposure as well as pre-event individual-level negative life events. In sum, while the media is an essential tool for keeping the populace informed in the aftermath of terrorism and other collective trauma, media outlets should consider the potential psychological ramifications of repeated exposure, and continue public discourse about how to address these issues.

## Data Availability Statement

The datasets presented in this article are not readily available because it should be requested to Santé publique France. Requests to access the datasets should be directed to Philippe Pirard, philippe.pirard@santepubliquefrance.fr.

## Ethics Statement

Ethical review and approval was not required for the study on human participants in accordance with the local legislation and institutional requirements. Written informed consent from the participants' legal guardian/next of kin was not required to participate in this study in accordance with the national legislation and the institutional requirements.

## Author Contributions

MR contributed to the data collection, design, data analysis, and writing of the manuscript. LS and DG contributed to the construction of the analyses, the interpretation of the findings, the writing of the manuscript, and were invited to contribute to this manuscript after the data collection was completed, and neither they nor their respective institutions contributed to the data collection. SV and YM contributed to the drafting of the manuscript. ER and PP contributed to the drafting of the manuscript and took the initiative to the data collection, and are, on behalf of the French National Agency for Public Health (Santé publique France), responsible for the ethical conduct of the study, and ensuring that it was conducted in accordance with the French legislation and institutional requirements. All authors contributed to discussions about the manuscript.

## Conflict of Interest

The authors declare that the research was conducted in the absence of any commercial or financial relationships that could be construed as a potential conflict of interest.
