# Supplementary material for: Media Exposure and Post-traumatic Stress Symptoms in the Wake of the November 2015 Paris Terrorist Attacks: A Population-Based Study in France
Source: Front Psychiatry. 2021 May 21;12:509457. doi: 10.3389/fpsyt.2021.509457 (PMC8175798; doi:10.3389/fpsyt.2021.509457)
Supplement: Supplementary file 1 [file Table_1.DOCX]

**APPENDIX**

| **Appendix 1: Sociodemographic characteristics of the BVA webpanel, the sample invited for study participation and the final study sample** | | | |
| --- | --- | --- | --- |
|  |  |  |  |
|  | **Total BCV panel ^a^** | **Sample invited for study participation^b^** | **Final study sample^c^** |
|  | % | % | % |
| **Sex** |  |  |  |
| Male | 35 | 42 | 48 |
| Female | 65 | 58 | 52 |
|  |  |  |  |
| **Age** |  |  |  |
| 15-24 years old | 9 | 26 | 15 |
| 25-34 years old | 23 | 24 | 15 |
| 35-49 years old | 39 | 30 | 25 |
| 50-64 years old | 22 | 14 | 24 |
| More than 65 years old | 7 | 6 | 21 |
|  |  |  |  |
| **Professional status** |  |  |  |
| Farmers | <0,5% | 1 | 1 |
| Craftspeople, shopkeepers, business owners | 4 | 5 | 4 |
| Executives, intellectual professions | 23 | 15 | 9 |
| Intermediate occupations | 7 | 16 | 14 |
| Employees | 30 | 5 | 17 |
| Workers | 4 | 10 | 13 |
| Retirees | 6 | 7 | 26 |
| Inactives | 26 | 31 | 16 |
|  |  |  |  |
| **Education level** |  |  |  |
| No degree | 12 |  | 10 |
| < High school diploma | 24 |  | 19 |
| High school diploma | 30 |  | 22 |
| > High school diploma | 34 |  | 49 |
|  |  |  |  |
| **Size of the urban area** |  |  |  |
| Rural districts | 19 |  | 23 |
| Between 2 000 and 20 000 residents | 14 |  | 18 |
| Between 20 000 and 100 000 residents | 11 |  | 13 |
| More than 100 000 residents | 31 |  | 30 |
| Parisian agglomeration | 25 |  | 16 |
| Note.  a: Characteristics presented are those of the nearly 700,000 individuals enrolled in the panel. Data are unweighted.  b: Characteristics presented are those of the 40,000 panelists contacted to integrate the study. Data are unweighted.  c: Characteristics presented are those of the 1,760 individuals enrolled in the survey. Those data are weighted.  Remarks: Unweighted characteristics of the 1,760 survey respondents remain confidential. Some information was not available (education level and size of the urban area for the 40,000 individuals contacted for the study). | | | |
